# Equine Hoof Stem Progenitor Cells (HPC) CD29 + /Nestin + /K15 + – a Novel Dermal/epidermal Stem Cell Population With a Potential Critical Role for Laminitis Treatment

**DOI:** 10.1007/s12015-021-10187-x

**Published:** 2021-05-26

**Authors:** Krzysztof Marycz, Ariadna Pielok, Katarzyna Kornicka-Garbowska

**Affiliations:** 1 International Institute of Translational Medicine (MIMT), ul. Jesionowa 11, 55-114 Malin Wisznia Mała, Poland; 2 Department of Experimental Biology, Wroclaw University of Environmental and Life Sciences, ul. CK Norwida 27, 50-375 Wrocław, Poland

**Keywords:** Stem cells, Hoof, Horse, Laminitis

## Abstract

Laminitis is a life threating, extremely painful and frequently recurrent disease of horses which affects hoof structure. It results from the disruption of blood flow to the laminae, contributing to laminitis and in severe separation of bone from the hoof capsule. Still, the pathophysiology of the disease remains unclear, mainly due to its complexity. In the light of the presented data, in the extremally difficult process of tissue structure restoration after disruption, a novel type of progenitor cells may be involved. Herein, we isolated and performed the initial characterization of stem progenitor cells isolated from the coronary corium of the equine feet (HPC). Phenotype of the cells was investigated with flow cytometry and RT-qPCR revealing the presence of nestin, CD29, and expression of progenitor cell markers including SOX2, OCT4, NANOG and K14. Morphology of HPC was investigated with light, confocal and SEM microscopes. Cultured cells were characterised by spindle shaped morphology, eccentric nuclei, elongated mitochondria, and high proliferation rate. Plasticity and multilineage differentiation potential was confirmed by specific staining and gene expression analysis. We conclude that HPC exhibit in vitro expansion and plasticity similar to mesenchymal stem cells, which can be isolated from the equine foot, and may be directly involved in the pathogenesis and recovery of laminitis. Obtained results are of importance to the field of laminitis treatment as determining the repairing cell populations could contribute to the discovery of novel therapeutic targets and agents including and cell‐based therapies for affected animals.

**Supplementary Information:**

The online version contains supplementary material available at 10.1007/s12015-021-10187-x.

## Introduction

One of the most debilitating diseases in horses since many years is laminitis – multifactorial disorder that is still poorly understood. During laminitis, serious pathological changes contribute to disruption of the distal phalangeal suspensory apparatus at the dermal–epidermal junction, which as a consequence destroys the lamellar structures [1]. The pathophysiology of laminitis includes sepsis related conditions which might be caused by overload with carbohydrates, abdominal surgery, gastrointestinal disease as well as non-septic endotoxemia that includes insulin resistance, Cushing syndrome or corticosteroid dysmetabolism. During laminitis, acute and chronic phases could be distinguished, that represents two clinical scenarios [2]. In acute phases, severe pain, lameness, and inflammation occur without radiological changes, while in chronic phase rotation of the third phalanx occurs causing detachment of lamina components leading to substantial damage of the basement membrane of the derma so that the structure collapses. Both acute and chronic phases of laminitis could have a poor prognosis and become the reason of equine disability and in consequence euthanasia. Thus, laminitis represents a serious medical event that has a strong socio-economic impact in the equine industry.

Laminitis is a complex disease that induces pathological changes at the meeting point of vascular system, inflammation, oxidative stress, and endocrinological system. Although many efforts has been paid to the development of effective therapeutic solutions for laminitis treatment, still no effective therapy is available [3–5]. Since laminitis includes three main molecular events including: inflammation, micromorphological and structural changes, stem cell based therapies seems to be a reasonable solution.

Mesenchymal stromal stem cells (MSCs), exhibit particular molecular and physiological features that allow to use them in clinical practice in many medical fields [6]. MSCs are plastic adherent cells that express the following surface markers CD105, CD44, CD29, CD73, CD90, and no expression of CD45, CD34, CD14, CD11b, or CD19, as well as multilineage differentiation potential [7]. MSC could give rise to multiple types of cell populations and therefore become a promising therapeutic agent [8]. However, the immunomodulatory and antiapoptotic effect of MSCs makes them even more promising when inflammatory-related injury and tissue regeneration are considered. Moreover, recent findings clearly indicate paracrine and autocrine activity of MSCs during various regenerative processes, which underlines their fundamental role in the initiation of so-called “regenerative process” [9–11]. Various sources of MSCs were in recent years were described including bone marrow-derived stem cells (BMSCs), adipose tissue-derived mesenchymal stem cells (ASCs), nail stem cells (NSCc) and many others [12, 13]. It seems that various stem cell pools possess unique properties which potentially could target specific injured tissue.

Therefore, bearing in mind the physiological nature of laminitis, here we identified novel MSCs subpopulation- CD29 + /Nestin + /K15 + HPC that were isolated from the equine coronary corium. These cells exhibited typical MSCs surface markers additionally extended to keratin 15 (K15), keratin 14 (K14), keratin 19 (K19), CD29, CD34, CD200, angiopoietin 1 (Ang1) and leucine-rich repeat-containing G protein-coupled receptor 6 (Lgr6) similarly to nail stem cells [12]. They multipotent nature as well as expressing markers of dermis and epidermis could in the near future become effectively applied during laminitis treatment. Here, for the first time we identified a novel stem cell pool of equine hooves identifying their surface markers as well as differentiation potential. Presented data shed a promising light on clinical application of HPC in the treatment of laminitis in the near future.

## Materials and Methods

### Tissue Harvest and Cell Culture

Samples of coronary corium tissue were collected post-mortem from 6 foals, at a local slaughterhouse. The animals were euthanized for reasons unrelated to this study. The tissue was dissected in sterile conditions with a scalpel blade and placed in Dulbecco’s modified Eagle’s medium/F12 (DMEM/F12, Sigma Aldrich/Merck, Poznan, Poland) supplemented with 1% penicillin/streptomycin mix (P/S, Sigma Aldrich, Munich, Germany).Within 1,5 h of harvest, EHSPC cells were isolated and cultured, following the protocol previously described by Yang et al. [14]. The osteogenic and chondrogenic differentiation were induced using StemPro™ Osteogenesis Differentiation Kit and StemPro™ Choondrogenesis Differentiation Kit(Thermo Fisher Scientific, Warsaw, Poland). Additionally, extracellular mineralization was visualized with Alizarin-Red staining, after 7 days of osteogenic differentiation. Safranin O stain was applied to confirm the formation of proteoglycans at day 7 and 14 of the chondrogenic differentiation. Images from cell culture were obtained using an inverted microscope Leica DMi1 integrated with camera MC170 (Leica Microsystems, KAWA.SKA Sp. z o.o., Zalesie Gorne, Poland). The proliferation rate of EHSPC cells was analysed using the MTS Assay Kit (ab197010, Abcam,Cambridge, UK), accordingly to the manufacturers protocol. The population doubling time was assessed using the reazurin-based assay kit (TOX -8) (Sigma Aldrich, Munich, Germany). All of the assays in this study were performed using EHSPC cells at maximum 3^rd^ passage.

### Cells Morphology Assessment

The morphology of EHSPC cells was visualized using a epifluorescent microscope (Zeiss, Axio Observer A.1) as well as confocal microscope (Leica TCSSPE, Leica Microsystems, KAWA.SKA Sp. z o.o., ZalesieGorne, Poland) and analyzed with Fiji is just ImageJ software (ImageJ 1.52n, Wayne Rasband, National Institute of Health, USA). The nuclei were stained with Hoechst stain(Thermo Fisher Scientific, Warsaw, Poland), accordingly to the manufacturers protocol. Additionally, MitoRed and Phalloidin-Atto 488 (Sigma Aldrich/Merck, Poznan, Poland) staining was performed, following established protocols [15]. Furthermore, cells were observed with a scanning electron microscope (SEM)( Evo LS 15 Zeiss). For the SEM imagining EHSPC cells were prepared as it was previously described [16].

### HE Staining

Fragments of fresh tissue were fixed in 4% Paraformaldehyde (PFA) solution for minimum 24 h. H&E staining was performed, using well established protocols. Briefly, after the incubation in the 4% PFA solution, tissue fragments were washed 3 times in Hank’s Balanced Salt Solution (HBSS) and dehydrated in a graded series of ethanol dilutions (from 50 to 96%, every 10%). Next, samples were first incubated in xylene (POCH SA—Avantor Performance Materials Poland SA) for 30 min and then moved into a xylen-parrafin solution(50% xylen, 50% paraffin) for 60 min at 37 °C. Subsequently, tissue fragments were placed in pure paraffin(Chempur, Poland) for 24 h at 65 °C. The paraffin was changed after 24 h, and the samples were incubated in pure paraffin at 65 °C for another hour. The tissue fragments were then embedded into paraffin blocks. Paraffin blocks were trimmed and cut into 6 μm slices, which were mounted onto glass slides. The hematoxylin and eosin staining (H&E) was conducted using Hematoxylin Solution (Harris Modified) and Eosin Y-solution 0.5% aqueous (Sigma Aldrich, Munich, Germany), respectively, accordingly to the manufacturers protocol. Lastly, the slides were sealed with DPX medium (Aqua-Med ZPAM – KOLASA sp.j, Łodź, Poland).

### Flow Cytometry

Flow Cytometry analyses were performed using S3e Cell Sorter (Bio-Rad, Hercules, CA, USA). In order to evaluate the expression of Nestin and CD29, cells were detached from the culture flask and washed 3 times, one time with HBSS supplemented with 2% FBS (Fetal Bovine Serum) and twice with pure HBSS. After each wash cells were centrifuged at 1200 g for 5 min. Subsequently, EHSPC cells were suspended in 300 μl of HBSS and incubated with PE Mouse anti-Nestin Clone 25/NESTIN antibody (BD Biosciences) and Alexa Fluor® 488 anti-human CD29 Antibody (BioLegend) for 30 min in dark, at the room temperature. Following the incubation, cells were centrifuged at 1200 g for 5 min, the supernatant was discarded and the cell pellet was suspended in 300 μl of HBSS for the flow cytometry analysis.

### RT and qPCR

Total RNA was isolated using the phenol–chloroform method [17]. The experimental cultures of EHSPC cells were homogenised with Extrazol® (Blirt DNA, Gdansk, Poland). The elimination of the genomic DNA (gDNA) from the samples was carried out using the DNase I from PrecisionDNAse kit (Primerdesign, BLIRT S.A, Gdansk, Poland). Following the purification, total RNA (500 ng) was transcribed into cDNA using Tetro cDNA Synthesis Kit (Bioline Reagents Limited, London, UK), according to the manufacturers protocol. The digestion of gDNA and the reverse transcription reaction (RT) were performed in the T100 Thermal Cycler (Bio-Rad, Hercules, CA, USA). Following the RT reaction, samples were used for qPCR analyses in order to analyse the expression of targeted genes. The specific primers used in the qPCR reaction are listed in Supplementary Table 1. All qPCR analyses were performed using the SensiFast SYBR & Fluorescein Kit (Bioline Reagents Ltd., London, United Kingdom) and the CFX Connect™ Real-Time PCR Detection System (Bio-Rad, Hercules, CA, USA). Reactions were performed in a 10-μL final volume. The analyses were performed at the following cycling conditions: 95 °C for 2 min, followed by 39 cycles at 95 °C for 15 s, annealing for 30 s, and elongation at 72 °C for 15 s. The obtained results were normalized to a reference gene expression- glyceraldehyde 3-phosphate dehydrogenase (GAPDH), the relative expression was calculated with the 2-ΔΔCQ method [18].

### Statistical Analysis

The data obtained in this study was analyzed with GraphPadPrism 5 software (La Jolla, CA, USA). Each time the mean was calculated from a minimum of three measurements. Differences between various groups were determined with one-way analysis of variance (ANOVA),parametric assays or the unpaired Student’s t–test. Differences with a probability of p ˂ 0.05 were considered as significant.

## Results

### Cells Isolation and Phenotyping

**Fig. 1:**
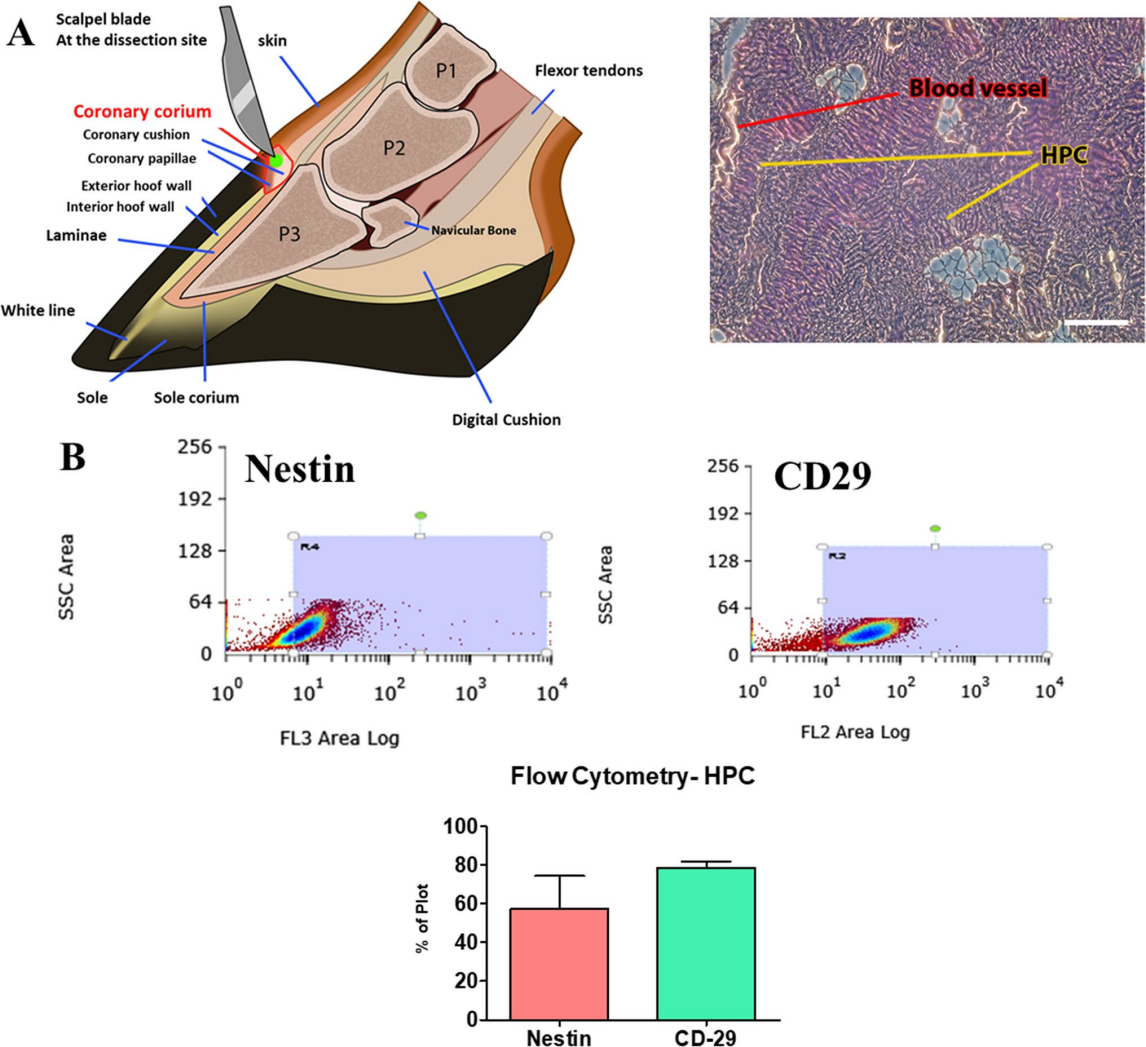
Cells isolation and phenotyping. Cells were isolated from coronary corium of horse feet (schematic drawing shows the cells niche). Tissue sections was harvested, fixed and subjected to H&E staining **A**. Isolated cells were cultured in vitro and subjected to flow cytometry analysis. Histograms and quantitative data show the results from nestin and CD29 analysis **B**. Furthermore, phenotype of cells was investigated with RT-qPCR analysis of Nestin **C**, K14 **D**, K15 **E**, VEGFA **F**, CD200 **G**, ANG1 **H**, OCT4 **I**, SOX2 **J** and NANOG **K** expression. Results expressed as mean ± SD. *p < 0.05, **p < 0.01, ***p < 0.001

**Fig. 2:**
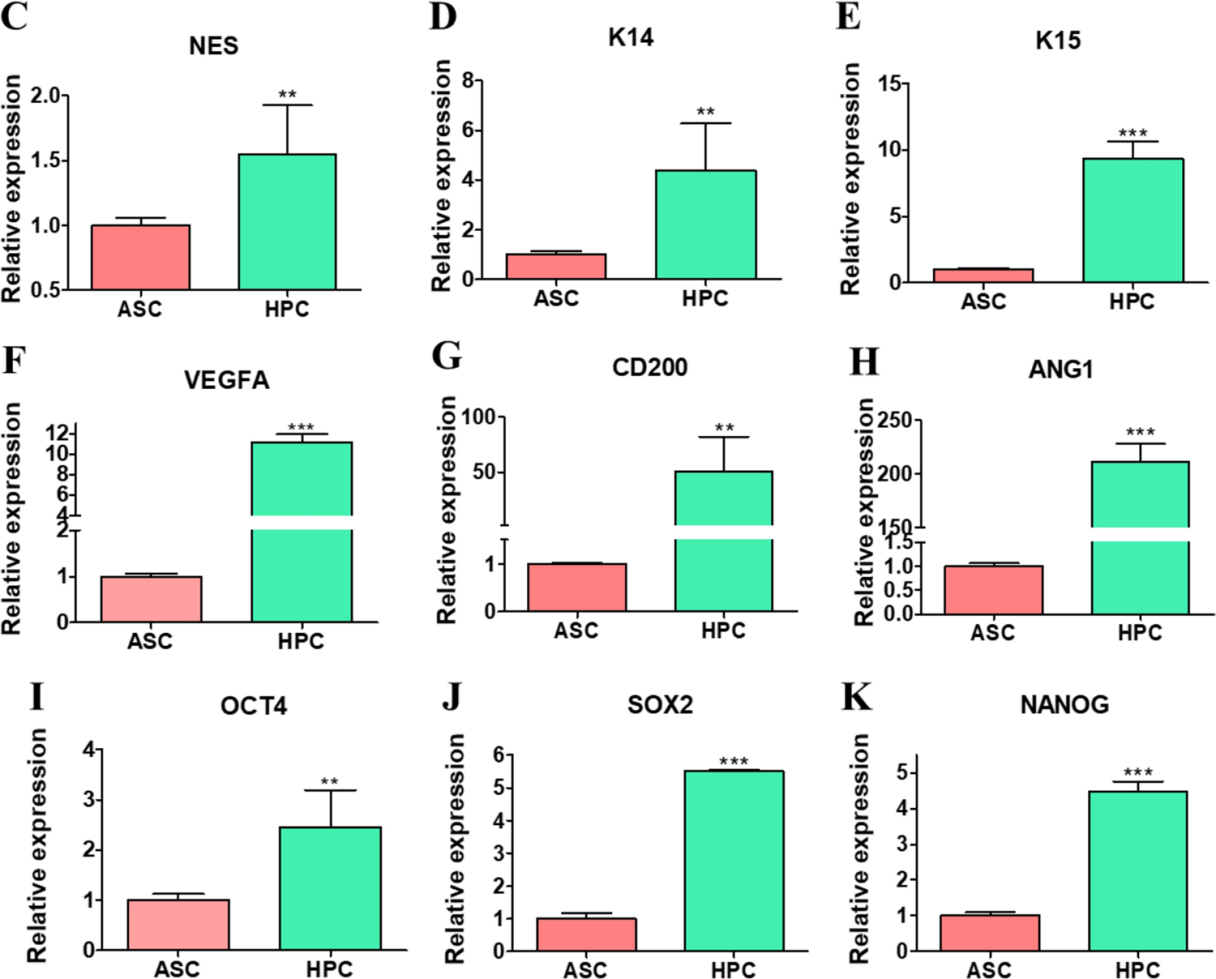
Morphology and proliferation. Fluorescent photomicrographs of cultured HPC visualised in epifluorescent **A** and confocal microscope **B**. Proliferation of cells was established with MTS in relation to ASC growth kinetics **C**. Based on obtained data, PDT was calculated **D**. Results expressed as mean ± SD. *p < 0.05, **p < 0.01, ***p < 0.001. Epifluorescent microscope magnification × 100, scale bar: 250 μm, confocal × 600, scale bar 10 μm

Light photomicrographs of H&E stainings of coronary corium from where cells were isolated (Fig. 1A). Passage 2 HPC cells were subjected to flow cytometry analysis which confirmed the expression of Nestin and CD29 (Fig. 2B). RT-qPCR analysis of isolated cells, confirmed the expression of Nestin (Fig. 1C), K14 (Fig. 1D), K15 (Fig. 1E), VEGFA (Fig. 1F), CD200 (Fig. 1G), ANG1 (Fig. 1H), OCT4 (Fig. 1I), SOX2 (Fig. 1J) and NANOG (Fig. 1K).

### Morphology and Proliferation

In order to visualise the morphology of isolated cells, they were subjected to fluorescent stainings. Nuclei were stained with DAPI while f-actin with phalloidin. Images were acquired with epifluorescent (Fig. 2A) and confocal microscope (Fig. 2B). Large, eccentric nuclei and elongated mitochondria evenly distributed within cytoplasm were observed. Proliferation of HPC of similar to ASC, however after 72 h, cells of hoof origin displayed increased growth kinetic (Fig. 2C). PDT of HPC was reduced while in comparison to ASC (Fig. 2D) which indicates on greatest proliferative potential of these cells.

### Multilineage Differentiation Potential

**Fig. 3:**
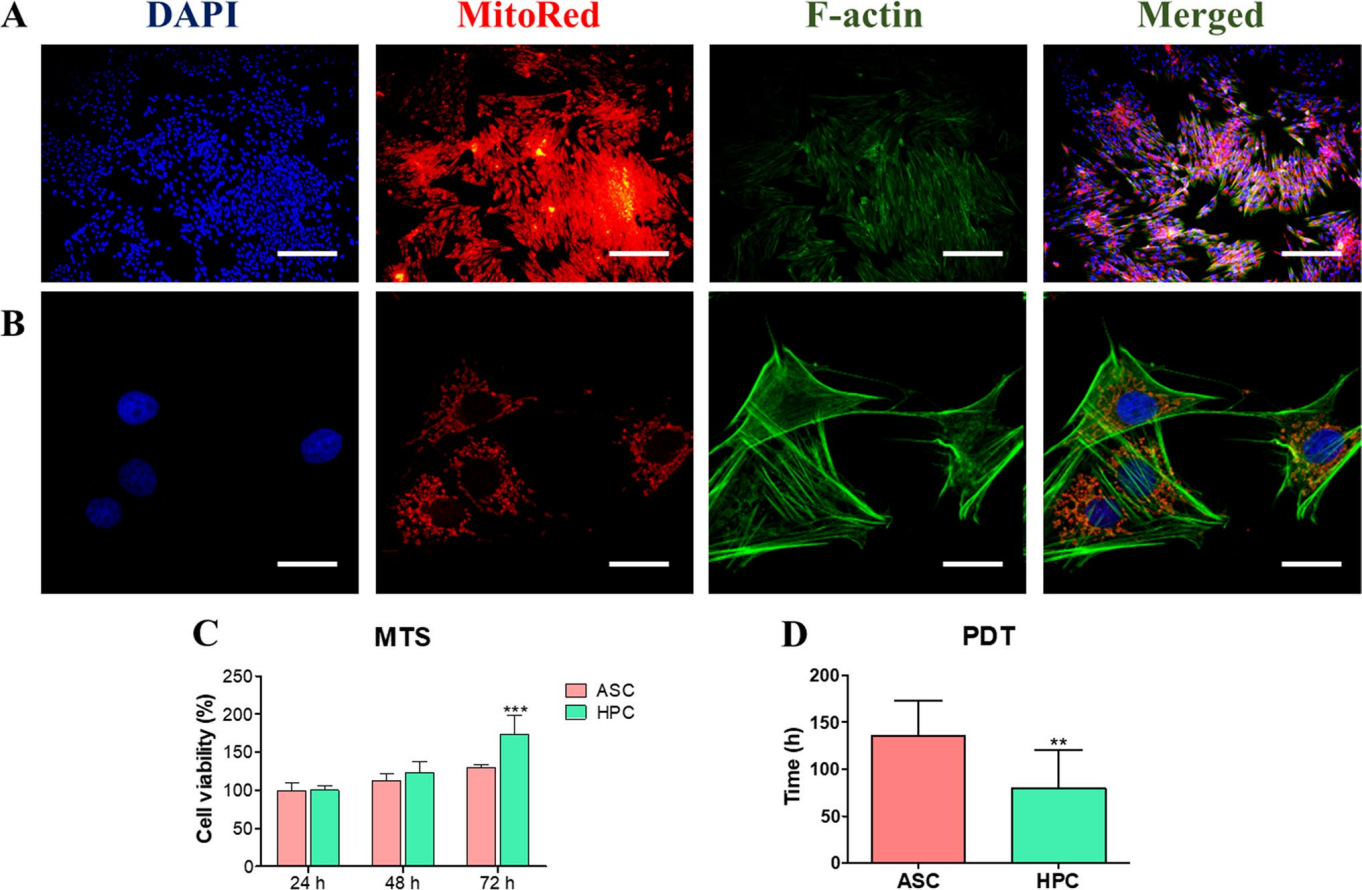
Multilineage differentiation potential of HPC. Photomicrographs of passage 1 cells cultured in control, chondrogenic and osteogenic medium **A**. Detailed morphology of cells after 14^th^ day of differentiation visualised with SEM **B**. Differentiation of cells after 7th day of culture was confirmed with Safranin O and Alizarin Red **C**. Expression of genes involved with differentiation process- COMP **D**, RUNX2 **E**, ACAN **F**, OPN **G**, RUNX3 (H), DCN **I**, COLL1 **J** and COLL2 **K** was investigated with RT-qPCR (CTRL- cells cultured in control medium, O- cells cultured in osteogenic and CH- cells cultured in chondrogenic differentiation media). Results expressed as mean ± SD. *p < 0.05, **p < 0.01, ***p < 0.001. Magnification (light microscope) × 100, scale bar: 250 μm

Passage 1 HPC were subjected for osteogenic and chondrogenic differentiation and their morphology was visualised with light, phase contrast microscope after 3rd, 7th and 14th day of culture (Fig. 3A). Cell cultured in control medium displayed spindle shaped morphology similar to progenitor cells including ASC. Cells cultured in differentiation medium underwent morphological change, become more cuboidal in shape, formed aggregates and extracellular matrix (ECM). Detailed morphology was visualised with SEM (Fig. 3B) and formation of ECM and bone modules was noted. Differentiation was further confirmed with specific stainings after 7th day (Fig. 3C). Safranin O revealed accumulation of proteoglycans during chondrogenesis while Alizarin Red stained mineralised matrix formed during osteogenic differentiation. What is more, the expression of marker genes after 14 day of differentiation was investigated with RT-qPCR in cells cultured in control (CTRL), osteogenic (O) and chondrogenic medium (CH). Expression of COMP (Fig. 3D), RUNX2 (Fig. 3E), ACAN (Fig. 3F), OPN (Fig. 3G), RUNX3 (Fig. 3H), DCN (Fig. 3I), COLL1 (Fig. 3J) and COLL2 (Fig. 3K) was investigated in relation to culture condition.

## Discussion

Equine adult stem progenitor cells of various origins are recently extensively used for the treatment of different disorders including musculoskeletal or endocrine systems [8, 19, 20]. The mesenchymal stem progenitor cells represent a unique population of stem cells that can be used for the treatment of particular disorders in horses, however, the efficacy of injured tissue treatment might strongly depend on their molecular phenotype and the place from which the stem cells were isolated. The tissue-specific microenvironment modulates stem cell fate and regenerative potential after their transplantation, which can suggest the clinical crosstalk between their place of origin and final tissue regeneration [21, 22].

Here, for the first time we identified a novel stem progenitor cell population that resides in the equine hoofs, particularly in coronary corium that could give rise to both: coronary dermis/epidermis as well as lamellar epidermis and finally the hoof wall. We speculate that this stem cell pool- HPC could play a protective function within the coronary corium, being capable of responding to coronary band injury, digital extensor tendons as well as hoof lamellae. For the first time, we identified octamer-binding transcription factor 4 (OCT4), sex determining region Y-box 2 (SOX2), CD105^+^, CD200^+^, K15^+^ and Ang1^+^ expressing HPC. Isolated cells are highly proliferative, plastic adherent and display fibroblast like morphology – all fundamental for confirmation of stemness. They also possess to multilineage differentiation potential as they were shown to give rise to mature and functional osteoblasts and chondroblasts.

In this study, we have identified that HPC express high levels of CD29, Nestin, K15, CD200, VEGF, and Ang1, which indicates on their potential role in the regeneration of dermal/epidermal and vascular injury during laminitis. CD200 has been shown to have immunomodulatory and immunosuppressive functions via apoptosis protecting the follicular stem cell niche from autoimmune attacks as well as modulating the local immune system [23]. In turn, both: vascular endothelial growth factor (VEGFA) and angiopoietin-1 (Ang1) have been proposed in horses to be a critical player in angiogenesis initiation by triggering endothelial cell proliferation, migration and neovascularization – all critical to regenerate the injured vascular system during laminitis [24]. Recent showed that Ang1 is critically involved in vascular and hematopoietic development as well as embryonic stem cell differentiation, mainly through its cognate receptor Tie2, which indicates on HPC involvement in the modulation of the new vascularization process [25]. Interestingly, when compared to ASCs, HPC were characterized by increased Ang1 expression which could suggest they advantage over ASCs during the vascularization process.

Here, we have found that HPC, when compared to widely investigated adipose derived stem progenitor cells (ASCs), possess significantly higher expression of keratin 15 (K15), suggesting their high potential for regeneration dermis/epidermis injure – that are a common events during laminitis. Cytokeratin 15 (clone C8/144B) has been shown to be a stem cell marker for hair follicle regeneration, since K15^+^ cells could be recruited to assist in epidermal re-epithelialization following injury [26]. Numerous adult epidermal stem cells have been shown to express K15 [12, 26–28] which suggests that identified by the us stem cell population could give rise a dermis/epidermis structure similar to human nail stem cells. Although previous excellent studies of Yang and Lopez [29] demonstrated and investigated the hoof progenitor stem cells isolated from the stratum lamellatum, that have both ectodermal (neurogenic) and mesodermal (osteogenic, adipogenic) differentiation abilities, we put our attention on HPC, since we hypothesize their localization as well as specific markers expression could be a beneficial for laminitis treatment.

Limitation of our research is that due to COVID-19 we did not have enough samples for performing more detailed cytometric analyses, immunomodulatory assays, or western blot which we could be the focus of the next research.

To summarize, here we identified a novel stem cell pool – OCT4, SOX2, CD105^+^, CD200^+^, K15 + , and Ang1^+^ HPC which meet the criteria of International Society of Cellular Therapy to be entitled stem cells and applied as a therapeutic agent. The HPC exhibit high pro-vasculogenic and immunosuppressive character, which shed a promising light for their future clinical application during laminitis.

## Supplementary Information

Below is the link to the electronic supplementary material.

Supplementary file1 (DOCX 13 KB)

## Data Availability

All datasets generated and/or analyzed during the current study are presented in the article, the accompanying Source Data or Supplementary Information files, or are available from the corresponding author upon reasonable request.
