# Supplementary material for: Equine Hoof Stem Progenitor Cells (HPC) CD29 + /Nestin + /K15 + – a Novel Dermal/epidermal Stem Cell Population With a Potential Critical Role for Laminitis Treatment
Source: Stem Cell Rev Rep. 2021 May 26;17(4):1478–85. doi: 10.1007/s12015-021-10187-x (PMC8149919; doi:10.1007/s12015-021-10187-x)
Supplement: Supplementary file 1 — Supplementary file1 (DOCX 13 KB) [file 12015_2021_10187_MOESM1_ESM.docx]

| **Primer** | **Forward sequence** | **Reverse seguence** |
| --- | --- | --- |
| **COLL-1** | GAAACTATCAATGGTGGTACCAAGT | AGCAGCCATCTACAAGAACAGT |
| **COLL-2** | AGGGAGACAGGGGTGATGTT | GGTCCAGTCTCTCCACGTTC |
| **OPN** | CATCGCCTATGCCCTTCCAG | TGTGTGGTCATGGCTTTCGT |
| **COPM** | AGTGTCGCAAGGATAACTGCGTGA | TCCTGATCTGTGTCCTTCTGGTCA |
| **RUNX-2** | ACTTTGCAGAGATGGGCCTC | CTAGGAAGTCGGGATGGGGA |
| **RUNX-3** | ATCAAGGTGACCGTGGATGG | GGAGAAAGGGTTCAGGTCCG |
| **ACAN** | CTAGAGGTCGTGGTGAAAGG | GTCGTAGGTCTCATTGGTGTC |
| **SOX9** | GAACGCCTTCATGGTGTGGG | TTCTTCACCGACTTCCTCCG |
| **DCN** | GATGCAGCTAGCCTGAGAGG | GTGTTGTATCCAGGTGGGCA |
| **Nestin** | ACTGAGAAGTTCCAGCTGGC | TCAGCCTCTAGAAGGGTCC |
| **K14** | TACGAGACGGAGCTGAACCT | TGGCCTCTCAGGCTATTCAT |
| **K15** | AACCAGGAGTACAAGACGCTG | AGAAACCACCTTTCCGTCCA |
| **CD-200** | GAGAGACTGGTGTTCAGGAGGT | GGTGTGTTCAGCAGCACTCTT |
| **VEGFA** | CCCACTGCGGAGTTCAACAT | TTTCTCCGCTCTGAGCAAGG |
| **ANG1** | AGGGTTTCCCAATTAGTCGCT | GCTTCGAGCCTGTAGCAGAT |
| **SOX2** | TTTCATGGTATGGCCCCAGG | GCTTCTCCGTCTCCGACAAA |
| **OCT4** | TCTCTTTGGGAAGGTGTTCAG | GTCTCAATACTAGTTCGCTTTCTC |
